# Reply: Choosing a stable housekeeping gene and protein is essential in generating valid gene and protein expression results

**DOI:** 10.1038/bjc.2011.36

**Published:** 2011-03-01

**Authors:** D Revaud, J Caradec, N Sirab, N Delacotte, S Loric

**Affiliations:** 1 Clinical Biochemistry and Genetics, Mondor University Hospital, Créteil, France

Sir,

We have read with a great interest the suggestion of Sullivan-Gunn *et al* (2011) concerning the measurement of the cDNA content with fluorescein-labelled oligonucleotides OliGreen (Invitrogen, Eragny, France), as a more reliable method than the use of housekeeping genes (HKGs) to normalise gene expression between biological samples in RT–QPCR assays. One of the main questions to address is effectively the relevance of the use of HKGs as an appropriate method to normalise RT–QPCR results.

Indeed, several studies, including ours, have demonstrated HKGs expression great variability according to experimental procedures or biological samples (de Kok *et al*, 2005; Caradec *et al*, 2010). All these observations raise the concept of finding the more stable HKG(s) for each experiment or study carried out before results normalisation. However, this search could become largely samples-, time- and money-consuming, leading to a large increase of the requested RT–QPCR assays to fulfil all the criteria needed to determine the best HKG or set of HKGs. As an example, we have undertaken gene expression study in four prostate cell lines PNT2, LNCaP, DU145 and PC3, in different cellular culture conditions. Among the 10 different internal control genes tested (ATP synthase subunit 6, *β*-actin, *β*-glucuronidase, *β*2 microglobuline, glyceraldehyde 3-phosphate dehydrogenase, transferine, hypoxanthine-guanine phosphoribosyltransferase, phosphoglycerokinase, TATA box binding protein and TAF7 TATA box binding protein factor), we found that the use of a set of three different HKGs (*β*-actin, ATP synthase subunit 6 and transferin) is essential to compare the gene expression in these four cell lines. This three HKGs expression analysis is already mandatory to complete this initial study, just restricted to normal control conditions. One would imagine that this set of HKGs has to be tested again in experimental conditions to define the more appropriate ones that have to be used when comparing cell lines in normal and experimental conditions.

In addition, data concerning HKGs expression variation studies and those dealing with the mean to achieve appropriate HKG(s) selection, such as the difference tolerated between HKGs’ crossing points (Cps) from considered samples, are not consistently published in details. These steps should be universally defined, as they are crucial to validate results and to draw conclusions on putative inter-laboratories variations.

Actually, we agree with the fact that cDNA measurement would certainly be a more reliable procedure to correctly normalise RT–QPCR results. The technique originally developed by Rhinn *et al* (2008), using fluorescence single-strand DNA detection with Oligogreen, seems a more specific way to normalise that seems not too difficult to implement in a clinical or research laboratory. Moreover, in contrast to another recent study suggesting the use of Agilent 2100 bioanalyzer technology with RNA 6000 LabChip to quantify cDNA concentration (Xing *et al*, 2009), the method described by Rhinn *et al* (2008) does not require neither RNAse H treatment nor cDNA purification steps, variable outputs of which could lead to misinterpretations in the result.

To sum up, the present remark of Sullivan-Gunn *et al* (2011) sounds really relevant and extends the already opened debate on the urgent need of an accurate and mandatory normalisation of RT–QPCR results, or more precisely standardisation of RT–QPCR technique, which will not necessarily be based on the use of HKGs.
